# C-C chemokine receptor type 5 activation stimulates adipocyte differentiation through ERK-dependent pathway

**DOI:** 10.7150/ijms.115524

**Published:** 2025-07-28

**Authors:** Luen-Kui Chen, Chien-Wei Chen, Shao-Yun Wu, Shui-Yu Liu, Liang-Yi Wu, Chi-Jen Chang, Leticia B. Sy, Chi-Chang Juan

**Affiliations:** 1 Institute of Physiology, College of Medicine, National Yang Ming Chiao Tung University, Taipei 112304, Taiwan.; 2 Department of Physical Education, Health, and Recreation, Teachers College, National Chiayi University, Chiayi 621302, Taiwan.; 3 Department of Bioscience Technology, College of Science, Chung-Yuan Christian University, Chung Li 32023, Taiwan.; 4 Division of Pediatric Surgery, Department of Surgery, Shin-Kong Wu Ho-Su Hospital, Taipei, Taiwan.; 5 School of Medicine, College of Medicine, Fu Jen Catholic University, New Taipei City, Taiwan.; 6 Department of Pediatrics, Cardinal Tien Hospital, New Taipei City 23148, Taiwan; 7 Department of Medical Research, Taipei Veterans General Hospital, Taipei 112201, Taiwan.

**Keywords:** adipocyte, differentiation, obesity, metabolic syndrome, RANTES, CCR5

## Abstract

Obesity is associated with low-grade chronic inflammation, and research has shown that RANTES and CCR5 mRNA levels are notably higher in the visceral adipose tissue of obese individuals compared to lean controls. However, the precise role of CCR5 activation in obesity development is still unclear. This study aims to explore the impact of CCR5 activation on adipogenesis and the underlying regulatory mechanisms. The study used 3T3-F442A preadipocytes and primary preadipocytes from wild-type (WT) and CCR5 knockout (CCR5^-/-^) mice to assess the role of CCR5 activation in adipocyte differentiation. To investigate the *in vivo* effects of CCR5 on obesity, male C57BL/6J WT and CCR5^-/-^ mice were fed either a normal chow (NC) or a high-fat diet (HFD) for two months. Plasma RANTES levels, fat pad weight, adipocyte size, and adipose CCR5 expression were measured. Treatment with RANTES resulted in increased intracellular triglyceride accumulation and enhanced expression of adipogenic transcription factors such as PPARγ, C/EBPα, and the adipocyte-specific protein aP2 during differentiation. These findings suggest that RANTES facilitates adipocyte differentiation. Moreover, pretreatment with the CCR5 inhibitor maraviroc and the ERK inhibitor PD98059 significantly reduced RANTES-induced adipocyte differentiation. RANTES also promoted differentiation in primary preadipocytes from WT mice, but not from CCR5^-/-^ mice. *In vivo*, WT mice on a high-fat diet showed higher plasma RANTES levels and increased adipose CCR5 expression, as well as obesity, whereas these changes were absent in CCR5^-/-^ mice. The results suggest that CCR5 activation by RANTES enhances adipocyte differentiation via an ERK-dependent pathway, and that CCR5 plays a critical role in the development of obesity.

## Introduction

Obesity is characterized by chronic, low-grade inflammation, marked by increased secretion of proinflammatory cytokines and the infiltration of immune cells into adipose tissue. It is a significant risk factor for conditions such as insulin resistance, type 2 diabetes mellitus, hypertension, dyslipidemia, non-alcoholic fatty liver disease, and cardiovascular disease. A study demonstrated the presence of CD3-positive T lymphocytes in human adipose tissue, along with increased expression of RANTES (regulated upon activation, normal T cell expressed and secreted, also known as CCL5) and its receptor CCR5 in the visceral adipose tissue of morbidly obese patients 1. Rausch's research found that CD8-positive lymphocytes were predominantly located in hypoxic regions within adipose tissue in obesity 2. Additionally, obesity is associated with an increased accumulation of T cells and macrophages in adipose tissue, which may significantly influence the functions of preadipocytes and adipocytes, thereby contributing to obesity-related diseases. RANTES, an adipokine, is upregulated in the adipose tissue of both obese mice and humans, with levels particularly elevated in male mice within the stromal/vascular fraction of white adipose tissue (WAT) compared to its adipocyte fraction 1. Furthermore, RANTES-induced T-cell chemotaxis can be neutralized by the addition of specific monoclonal antibodies to media conditioned by WAT isolated from obese male mice. These findings highlight the role of RANTES-induced T-cell chemotaxis in the context of obese WAT 3.

Several lines of evidence indicate that CCR5 and its ligands (CCL3, CCL4, and RANTES) play a crucial role in regulating the inflammatory response. For instance, CCR5 exacerbates inflammation in mouse adipose tissue by influencing macrophage recruitment and the switching between M1 and M2 phenotypes, contributing to insulin resistance and obesity 4. Our research has shown that inhibiting CCR5 or CCR5 deficiency can reduce obesity and inflammation induced by a high-fat diet (HFD) 5. Ota's literature review also highlights CCR5 as a novel link between obesity and inflammation in adipose tissue 6. These findings suggest that CCR5 signaling plays a significant role in the development of obesity; however, the specific mechanisms through which CCR5 connects obesity and inflammation remain unclear.

In general, obesity is characterized by excessive fat accumulation due to an imbalance between energy intake and expenditure 7. High energy intake and/or low energy expenditure can lead to the development of obesity. From the perspective of energy metabolism, CCR5 signaling may alter adipose function by regulating adipose development. Therefore, it is essential to evaluate the mechanisms through which CCR5 activation influences adipocyte differentiation. Based on these observations, this study aims to investigate the role of CCR5 activation in adipogenesis and its underlying regulatory mechanisms. We found that CCR5 expression increased during adipocyte differentiation, and its activation enhanced this process via an ERK-dependent pathway. Additionally, CCR5 deficiency significantly reduced HFD-induced obesity in mice.

## Materials and Methods

### Materials

Dulbecco's Modified Eagle Medium (DMEM), penicillin, and streptomycin were obtained from Gibco BRL (Gaithersburg, MD, USA). Fetal Bovine Serum (FBS) was sourced from Biowest (Nuaillé, France). Isobutylmethylxanthine (IBMX), dexamethasone, and other chemicals were purchased from Sigma-Aldrich (St. Louis, MO, USA). Antibodies against C/EBPα, PPARγ, and β-actin were acquired from Santa Cruz Biotechnology (Santa Cruz, CA, USA), while antibodies targeting aP2 were obtained from Chemicon International (Temecula, CA, USA). The triglyceride assay kit was procured from DiaSys Diagnostic Systems GmbH (Holzheim, Germany), and mouse RANTES was sourced from ProSpec-Tany Techno Gene Ltd. (Ness Ziona, Israel). Maraviroc was obtained from Tocris Bioscience (Minneapolis, MN, USA), and PD98059 and SB203580 were from Enzo Life Science Inc. (Farmingdale, NY, USA).

### Cell Culture and Differentiation Induction

Murine 3T3-F442A preadipocytes, generously provided by Howard Green from Harvard University (Cambridge, MA, USA), were seeded onto 60-mm dishes or 12-well plates (Falcon®, Becton Dickinson, NJ, USA) and cultured in growth medium (DMEM supplemented with 100 units/mL of penicillin, 100 µg/mL of streptomycin, and 10% FBS) in a 10% CO_2_ atmosphere until confluence. After reaching confluence, cells were treated with differentiation medium (DMEM containing 100 units/mL penicillin, 100 µg/mL streptomycin, 1 U/mL insulin, and 10% FBS), with medium changes every three days until full differentiation was achieved. Successful differentiation was confirmed by the accumulation of lipid droplets, assessed via oil red O staining.

The immortalized mouse epididymal preadipocyte cell line, a gift from Johannes Klein's laboratory, was generated as previously described 8. Cells were grown to confluence (day 0) in differentiation medium (DMEM with 20% FBS, 100 units/mL penicillin, 100 µg/mL streptomycin, 20 nM insulin, and 1 nM T3). Adipocyte differentiation was induced by treating confluent cells for 48 hours with induction medium (DMEM containing 20% FBS, 100 units/mL penicillin, 100 µg/mL streptomycin, 20 nM insulin, 1 nM T3, 0.5 mM IBMX, 0.5 mM dexamethasone, and 0.125 mM indomethacin). After this period, cells were returned to differentiation medium, which was changed every two days. Differentiated cells were collected on day 10 for subsequent experiments.

### Isolation and Differentiation of Primary Stromal Vascular Fraction

Eight-week-old male C57BL/6J mice were used to isolate primary preadipocytes, following previously described methods with minor modifications. The epididymal fat pads were excised, minced, and digested with collagenase before being filtered through a 100-μm nylon mesh. The pellet containing the stromal vascular fraction (SVF) was cultured in DMEM supplemented with 20% FBS, 100 units/mL penicillin, and 100 µg/mL streptomycin. Cells were grown until 12-24 hours post-confluence in differentiation medium (DMEM with 20% FBS, 100 units/mL penicillin, 100 µg/mL streptomycin, 20 nM insulin, and 1 nM T3). Adipocyte differentiation was induced for 48 hours with induction medium (DMEM containing 20% FBS, 100 units/mL penicillin, 100 µg/mL streptomycin, 20 nM insulin, 1 nM T3, 0.5 mM IBMX, 0.5 mM dexamethasone, and 0.125 mM indomethacin). Following induction, cells were returned to differentiation medium, which was changed every two days. Differentiated cells were collected on day 10 for further analysis.

### Triglyceride Measurement

Adipocytes were disrupted by sonication, and triglyceride levels were measured using commercial kits according to the manufacturer's instructions (DiaSys Diagnostic Systems GmbH & Co. KG, Holzheim, Germany).

### Oil Red O Staining

To assess lipid accumulation, cells cultured in 12-well plates were washed with PBS and fixed with 4% paraformaldehyde at room temperature. They were then stained with a 60% filtered Oil Red O stock solution (0.3 g/100 mL isopropanol). The stained cells were washed once with 60% (v/v) isopropanol and twice with distilled water. Lipid accumulation was quantified by extracting the Oil Red O from stained cells with isopropanol, and the optical density was measured at 510 nm using a plate reader 9.

### Immunoblotting

Protein levels were determined by western blotting as described previously. Briefly, cells were lysed with a buffer containing 1% Triton X-100, 50 mM KCl, 25 mM HEPES (pH 7.8), 10 µg/mL of leupeptin, 20 µg/mL of aprotinin, 125 µM dithiothreitol, 1 mM phenylmethylsulfonyl fluoride, and 1 mM sodium orthovanadate. Protein concentration was measured using a Bradford protein assay kit (Bio-Rad Laboratories, Inc., Hercules, CA, USA). Samples (30 µg of total protein) in 50 µL of reducing sample buffer were boiled for 5 minutes, resolved on SDS polyacrylamide gels, and transferred onto PVDF membranes. The membranes were pre-blocked with 5% dry milk at room temperature for 2 hours, incubated with primary antibody overnight at 4°C, and then with a peroxidase-conjugated secondary antibody (Sigma-Aldrich Corp., St. Louis, MO, USA) for 1 hour at room temperature. Protein expression levels were detected using a chemiluminescence reagent (Amersham Biosciences, GE Healthcare, Bucks, UK).

### HFD-Induced Obesity in Mice

All animal experiments were conducted in accordance with the guidelines on animal care including the ARRIVE guidelines and use as approved by the Institutional Animal Care and Use Committee of National Yang Ming Chiao Tung University (IACUC No. 1100519). Male C57BL/6 (WT) mice were purchased from the National Laboratory Animal Center (Taipei, Taiwan), while male CCR5^-/-^ mice (Ccr5^tm1Kuz/J^) were acquired from the Jackson Laboratory (Bar Harbor, ME). CCR5^-/-^ mice were backcrossed to C57BL/6 mice for at least ten generations to ensure a similar genetic background to WT mice. Mice were housed in microisolator cages under controlled conditions: 24±1 °C temperature, 60% humidity, and a 12 h:12 h light/dark cycle. They had ad libitum access to either normal chow diet (LabDiet® 5001, 3.02 kcal/g) or a high-fat diet (HFD; Envigo TD.06414, 60% kcal from fat, 5.1 kcal/g), as well as sterile water. The experiment lasted for three months, then the mice were euthanized using excess CO₂. The body weight, fat pad weight, adipocyte size and distribution, adipose CCR5 expression, and serum RANTES levels were measured.

### Hematoxylin and Eosin (H&E) and Immunohistochemistry (IHC) Staining of Adipose Tissue

Epididymal adipose tissues were fixed in 4% paraformaldehyde overnight at 4 °C, embedded in paraffin, and sectioned at 6 μm thickness. For H&E staining, sections were deparaffinized, rehydrated, stained with hematoxylin and eosin, dehydrated, and mounted. Images were captured using a light microscope, and adipocyte size was quantified using ImageJ software. At least 100 adipocytes per mouse were analyzed to determine average diameter and cumulative size distribution. Adipocyte number per mouse was estimated based on fat pad weight and average cell volume. For IHC, sections underwent antigen retrieval in citrate buffer (pH 6.0), followed by blocking in 5% BSA and incubation with anti-F4/80 antibody (1:100, BioLegend) overnight at 4 °C. Sections were then incubated with HRP-conjugated secondary antibody, developed with DAB, and counterstained with hematoxylin. F4/80-positive area was quantified using ImageJ software.

### Quantitative Real-Time PCR (qPCR)

Total RNA was extracted from epididymal adipose tissue using the RNeasy Plus Mini Kit (Qiagen), and cDNA was synthesized using the RevertAid First Strand cDNA Synthesis Kit (Thermo Fisher Scientific) according to the manufacturers' protocols. qPCR was conducted using TaqMan Gene Expression Assays and FastStart Universal Probe Master Mix (Applied Biosystems) on a QuantStudio 3 system. The following TaqMan probes were used: Adgre1 (F4/80): Mm00802529_m1; Ccl2 (MCP-1): Mm00441242_m1; Nos2 (iNOS): Mm00440502_m1; Ccr5: Mm01963251_s1; Ccl5: Mm01302427_m1. Gapdh (endogenous control): Mm99999915_g1. Gene expression was normalized to Gapdh and quantified using the ΔΔCt method.

### Statistical Analysis

Experiments were repeated three times, and results are expressed as mean ± SEM. For comparisons between two groups, an unpaired Student's t-test was used. For comparisons between multiple groups, statistical significance was determined using one-way or two-way analysis of variance (ANOVA). P value < 0.05 was considered to be statistically significant.

## Results

### Expression of CCR5 During Adipocyte Differentiation

**Figure 1:**
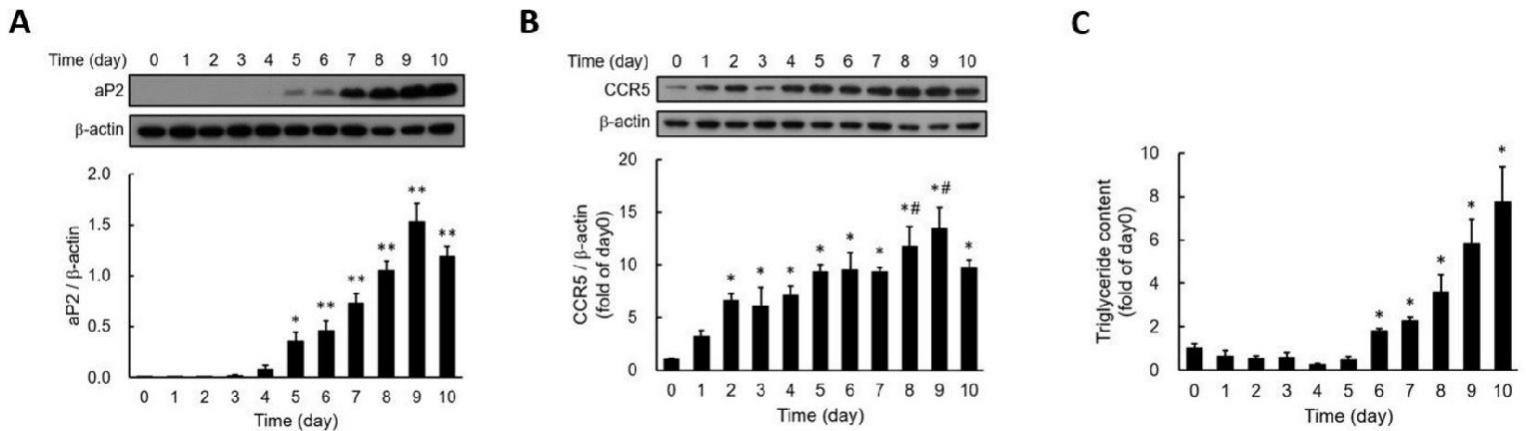
**Expression of aP2 (A) and CCR5 (B) and the accumulation of triglycerides (C) during the differentiation of 3T3-F442A preadipocytes are shown.** Western blot analysis was performed to assess the expressions of aP2 **(A)** and CCR5 **(B)** during the differentiation process. Triglyceride accumulation was measured using a triglyceride assay kit **(C)**. Statistical values are expressed as mean ± SEM. A significant difference compared to Day 0 is indicated by *p < 0.05, and differences compared to Day 0 are also indicated by #p < 0.05 (n = 3).

To investigate the involvement of CCR5 in adipocyte differentiation, we examined changes in CCR5 expression during the differentiation of 3T3-F442A adipocytes. Fatty acid binding protein 4 (FABP4), also known as adipocyte protein 2 (aP2), is widely recognized as a marker for differentiated adipocytes. We found that aP2 expression became significantly detectable by day five post-differentiation, peaking on day nine (Fig. 1A). CCR5 expression was also detectable from day two (Fig. 1B). Additionally, triglyceride accumulation showed a significant increase by day six and continued to rise through day ten (Fig. 1C). These results indicate that the expression of aP2 and CCR5, as well as lipid accumulation during adipocyte differentiation, is time-dependent, with CCR5 expression increasing early in the differentiation process.

### RANTES Stimulates Adipocyte Differentiation

**Figure 2:**
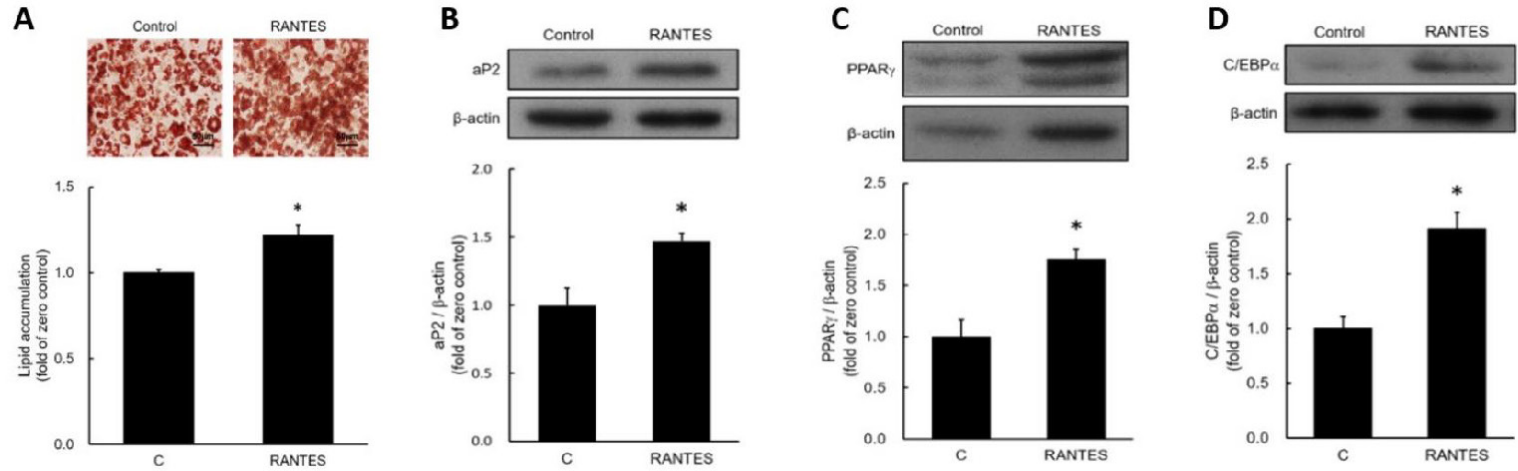
**Effects of CCR5 activation on lipid droplet accumulation in adipocytes.** Post-confluent 3T3-F442A preadipocytes were induced to differentiate in the presence or absence of various concentrations (10^-10^ M) of RANTES. After 10 days of differentiation, triglyceride accumulation was assessed using Oil Red staining (upper panel) and a triglyceride assay kit (lower panel) **(A)**, along with Western blot analysis of aP2 expression **(B)** during the differentiation of 3T3-F442A preadipocytes. After 3 days of differentiation, Western blot analysis was performed to evaluate PPARγ **(C)** and C/EBPα **(D)** expressions during the differentiation process. Statistical values are expressed as mean ± standard error of the mean (SEM). A significant difference compared to the control group is indicated by *p < 0.05 (n = 3).

To assess the effects of RANTES on adipocyte differentiation, post-confluent preadipocytes were induced to differentiate in the presence or absence of 10^-10^ M RANTES. After ten days, triglyceride accumulation was measured using oil red O staining and a triglyceride assay kit. The results indicated that RANTES treatment significantly enhanced lipid accumulation in adipocytes compared to the control group (Fig. 2A). Moreover, the expression of the adipocyte-specific protein aP2 was elevated in the RANTES-treated group compared to controls (Fig. 2B). We also analyzed the expression of PPARγ and C/EBPα at day 3 during the early stages of differentiation, finding that RANTES treatment stimulated the expression of both PPARγ (Fig. 2C) and C/EBPα (Fig. 2D) compared to the untreated controls. Collectively, these findings suggest that RANTES promotes adipocyte differentiation.

### CCR5 Antagonist Maraviroc Blocks RANTES-Stimulated Adipocyte Differentiation

**Figure 3:**
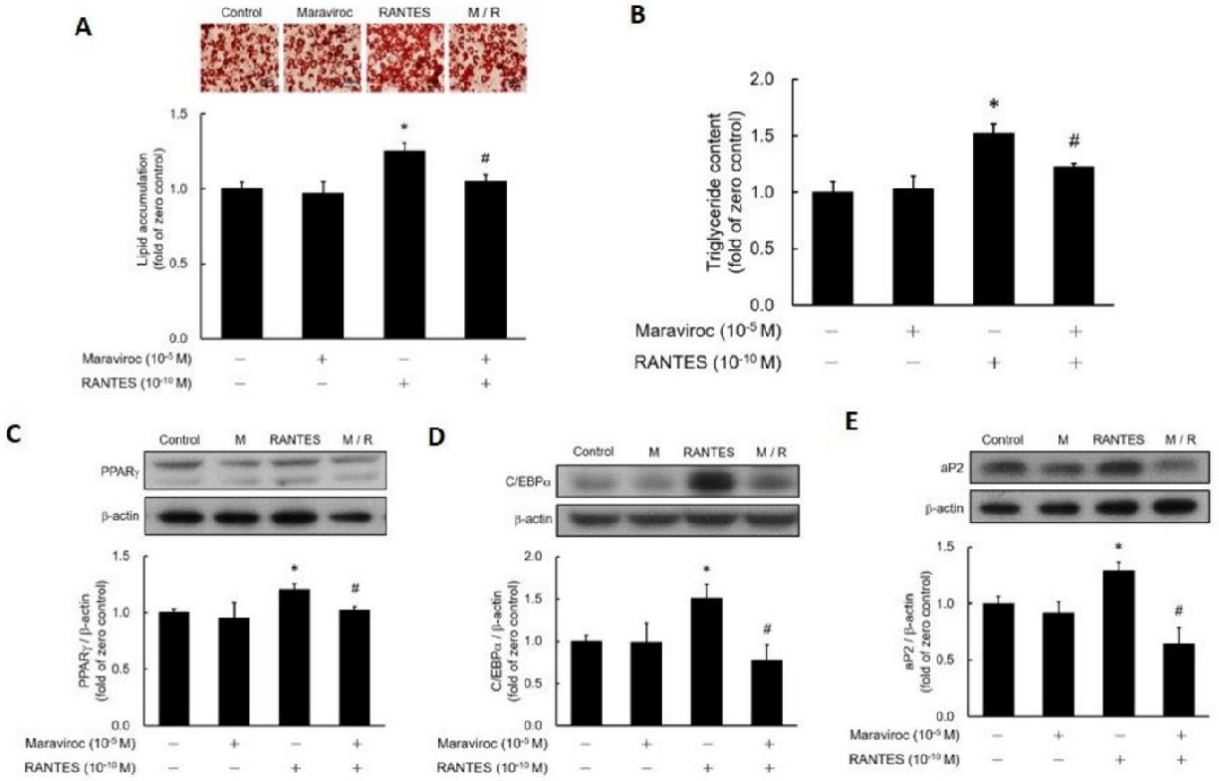
** The effect of the CCR5 competitive antagonist (Maraviroc) on the differentiation of adipocytes promoted by RANTES.** 3T3-F442A preadipocytes were treated with Maraviroc (10^-5^ M) for 1 hour prior to the initiation of their differentiation on Day 0, followed by treatment with RANTES (10^-10^ M) for three days. On Day 10 of cell differentiation, cell homogenates were collected, and triglyceride accumulation was determined using Oil Red staining **(A)** and a triglyceride assay kit **(B)**. After 3 days of differentiation, Western blot analysis was conducted to assess PPARγ **(C)** and C/EBPα **(D)** expressions. On Day 10, Western blot analysis of aP2 **(E)** expression was also performed. Statistical values are expressed as mean ± SEM. A significant difference compared to the untreated group is indicated by *p < 0.05; a significant difference compared to the group treated with RANTES alone is indicated by #p < 0.05 (n = 3).

To determine whether RANTES-induced adipocyte differentiation occurs via CCR5 activation, post-confluent preadipocytes were pretreated with or without the CCR5 antagonist Maraviroc before assessing the impact of RANTES on differentiation. Results showed that Maraviroc pretreatment effectively blocked RANTES-stimulated lipid accumulation (Fig. 3A, 3B). Additionally, the RANTES-induced expressions of PPARγ (Fig. 3C), C/EBPα (Fig. 3D), and aP2 (Fig. 3E) were significantly inhibited by Maraviroc pretreatment. Thus, these results confirm that RANTES stimulates adipocyte differentiation through CCR5 activation.

### RANTES Stimulates Adipocyte Differentiation via an ERK-Dependent Pathway

**Figure 4:**
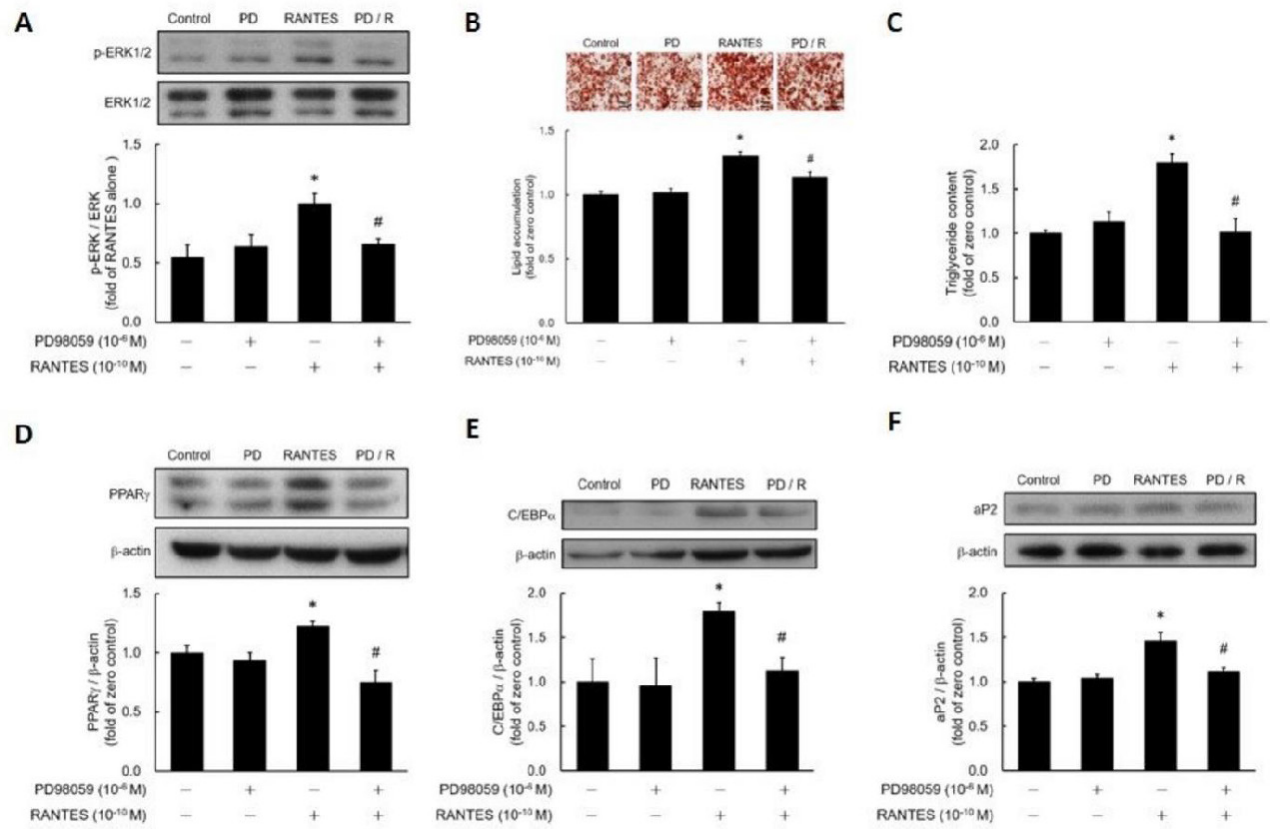
** The effect of the ERK1/2 inhibitor (PD98059) on the differentiation of adipocytes promoted by RANTES.** 3T3-F442A preadipocytes were treated with the ERK1/2 inhibitor (PD98059) for 1 hour before treatment on Day 0 of their differentiation process, followed by treatment with RANTES (10^-10 M) for three days. On Day 0, cells were treated with RANTES (10^-10^ M) for a short duration of 15 minutes, and Western blot analysis was performed to assess the phosphorylation of ERK1/2 **(A)**. On Day 10 of differentiation, triglyceride accumulation was measured using Oil Red staining **(B)** and a triglyceride assay kit **(C)**. After 3 days of differentiation, Western blot analysis of PPARγ **(D)** and C/EBPα **(E)** expressions was conducted. On Day 10, Western blot analysis of aP2 **(F)** expression was also performed. Statistical values are expressed as mean ± SEM. A significant difference compared to the untreated group is indicated by *p < 0.05; a significant difference compared to the group treated with RANTES alone is indicated by #p < 0.05 (n = 3).

To assess whether RANTES promotes ERK phosphorylation in preadipocytes, post-confluent preadipocytes were treated with RANTES, and ERK phosphorylation was analyzed using western blotting. The results demonstrated that RANTES significantly stimulated ERK phosphorylation, which could be effectively blocked by the ERK inhibitor PD98059 (Fig. 4A). To investigate if RANTES promotes adipocyte differentiation through the ERK-dependent pathway, post-confluent preadipocytes were pretreated with or without PD98059 before assessing the effect of RANTES on differentiation. The findings indicated that PD98059 pretreatment successfully prevented RANTES-induced lipid accumulation (Fig. 4B, 4C). Furthermore, RANTES-stimulated expression of PPARγ (Fig. 4D), C/EBPα (Fig. 4E), and aP2 (Fig. 4F) were also significantly inhibited by PD98059 pretreatment. Therefore, RANTES stimulates adipocyte differentiation through an ERK-dependent pathway.

### Effects of RANTES on Differentiation of Primary Preadipocytes Isolated from CCR5 Knockout (CCR5^-/-^) and Wild-Type (WT) Mice

**Figure 5:**
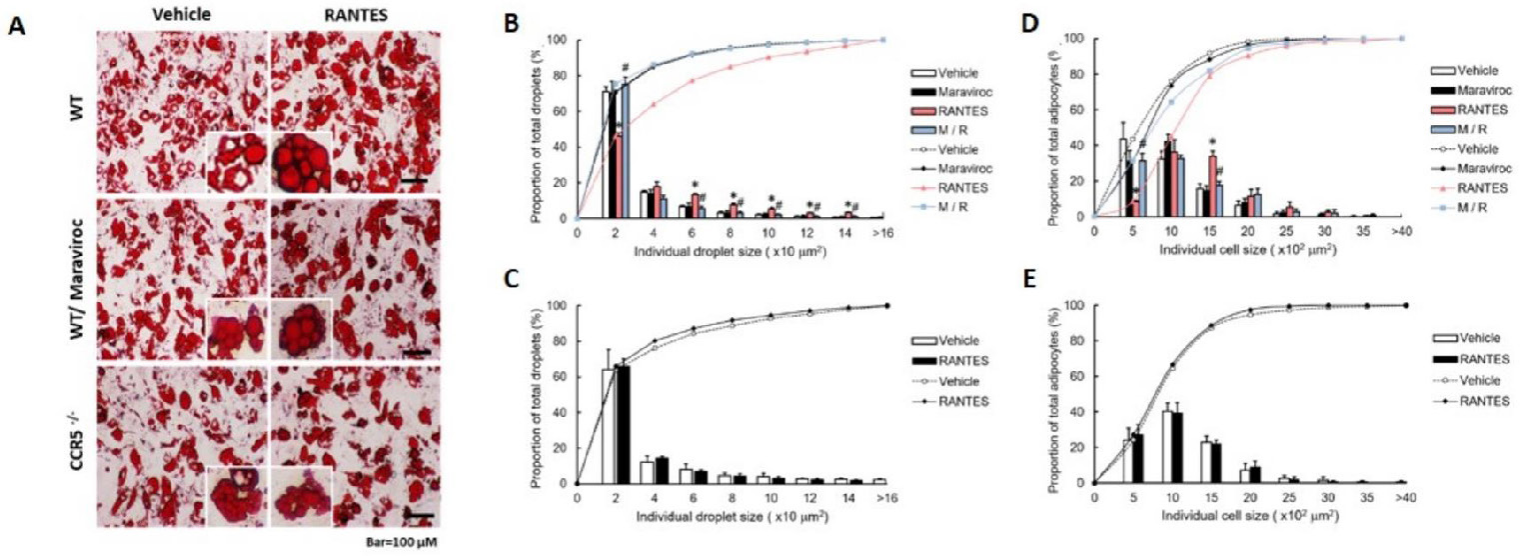
** The effect of RANTES and the CCR5 competitive antagonist (Maraviroc) on primary mouse preadipocytes from WT and CCR5^-/-^ mice.** Primary WT preadipocytes **(B, D)** were treated with Maraviroc (10^-5^ M) for 1 hour before treatment on Day 0 of their differentiation process, followed by treatment with RANTES (10^-10^ M) for three days. **(A)** On Day 10, cells were analyzed for oil droplet accumulation using the Oil Red staining method, and the staining results were observed under a microscope. **(B, C)** ImageJ software was used to compare the cell size distribution and analyze the differences in the percentage of cell sizes. **(D, E)** ImageJ software was also used to compare the lipid droplet distribution and analyze the differences in the percentage of lipid droplet sizes. Statistical values are expressed as mean ± standard error of the mean (SEM). A significant difference compared to the untreated group within the same category is indicated by *p < 0.05; a significant difference compared to the group treated with RANTES alone is indicated by #p < 0.05 (n = 3).

We further validated the effect of RANTES-induced CCR5 activation on the differentiation of primary preadipocytes isolated from CCR5^-/-^ and WT mice. Primary preadipocytes were pre-treated with or without Maraviroc, and RANTES-induced adipocyte differentiation was assessed through lipid droplet accumulation. Oil red O-stained images of adipocytes are shown in Figure 5A. The results indicated that the size distribution curve of adipocytes in the RANTES-treated group shifted significantly to the right compared to the vehicle group, with Maraviroc pretreatment significantly suppressing this effect (Fig. 5D). To analyze the size distribution of lipid droplets, we observed a similar significant rightward shift in the RANTES-treated group compared to the vehicle group, which was also significantly inhibited by Maraviroc pretreatment (Fig. 5B). In primary preadipocytes isolated from CCR5^-/-^ mice, the effects of RANTES on adipocyte differentiation were largely abolished (Fig. 5C, 5E). Collectively, these findings suggest that RANTES enhances the size of adipocytes and intracellular lipid droplets during the differentiation of primary preadipocytes, indicating that CCR5 activation is crucial for regulating adipogenesis.

### Deficiency of CCR5 Ameliorates HFD-Induced Obesity in Mice

**Figure 6:**
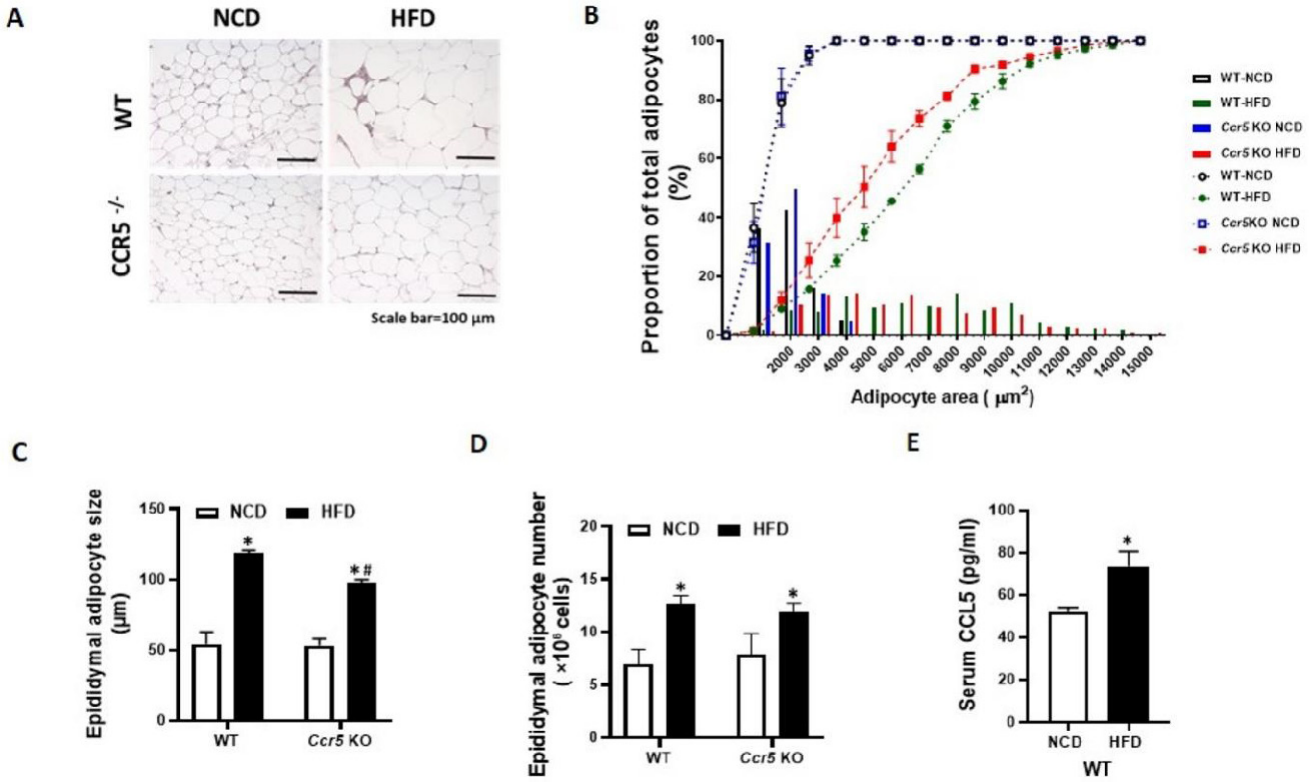
** CCR5-deficient mice exhibit reduced HFD-induced adipocyte hypertrophy.** Mice from each genotype were fed either a normal chow diet (NCD) or a high-fat diet (HFD) starting at 8 weeks of age for a total duration of 12 weeks (n = 6). **(A)** Representative H&E staining of epididymal fat, with a scale bar indicating 100 µm. **(B)** Cumulative distribution curves of adipocyte area, showing a rightward shift in WT-HFD mice indicative of adipocyte hypertrophy, while Ccr5⁻/⁻-HFD mice showed a leftward shift toward smaller adipocyte sizes. **(C)** Quantification of epididymal adipocyte cell size from WT and CCR5^-/-^ mice. **(D)** Total adipocyte number in epididymal fat, expressed as ×10⁶ cells. **(E)** Circulating RANTES levels. Data are presented as mean ± SEM. *p < 0.05 (NCD vs. HFD); #p < 0.05 (CCR5-deficient mice vs. wild-type mice).

CCR5^-/-^ and WT mice were fed a high-fat diet (HFD) for 12 weeks, during which we evaluated changes in body weight, epididymal fat pad weight, adipose CCR5 expression, circulating RANTES levels, and adipocyte size. Results indicated that 12 weeks of HFD feeding significantly increased body weight, fat pad weight, and circulating RANTES levels in WT mice (Table 1). In contrast, HFD-induced obesity was significantly mitigated in CCR5^-/-^ mice. Additionally, adipocyte size from the HFD group was significantly larger compared to that from the control group in WT mice (Fig. 6 A, 6C). HFD-induced adipocyte hypertrophy in WT mice was reduced in CCR5^-/-^ mice. To further analyze the adipocyte size distribution across the four groups, the results showed that the size distribution curve of adipocytes was significantly shifted to the right in the HFD group compared to the control group in WT mice (Fig. 6B). In the CCR5^-/-^ mice, the size distribution curve of adipocytes in the HFD group was significantly shifted to the left compared to that in the WT HFD group. Quantification of total adipocyte number per mouse indicated that HFD significantly increased adipocyte number in both WT and CCR5⁻/⁻ mice relative to their respective NCD controls (Fig. 6D). Although CCR5⁻/⁻-HFD mice exhibited a slightly higher adipocyte number than WT-HFD mice, this difference was not statistically significant. Lastly, serum CCL5 levels were significantly elevated in WT mice following HFD feeding (Fig. 6E), indicating diet-induced activation of the CCL5-CCR5 axis. These *in vivo* observations suggest that CCR5 deficiency protects against overnutrition-induced adipogenesis, underscoring the important role of CCR5 in this process.

### CCR5 deficiency attenuates adipose tissue inflammation in HFD-fed mice

**Figure 7:**
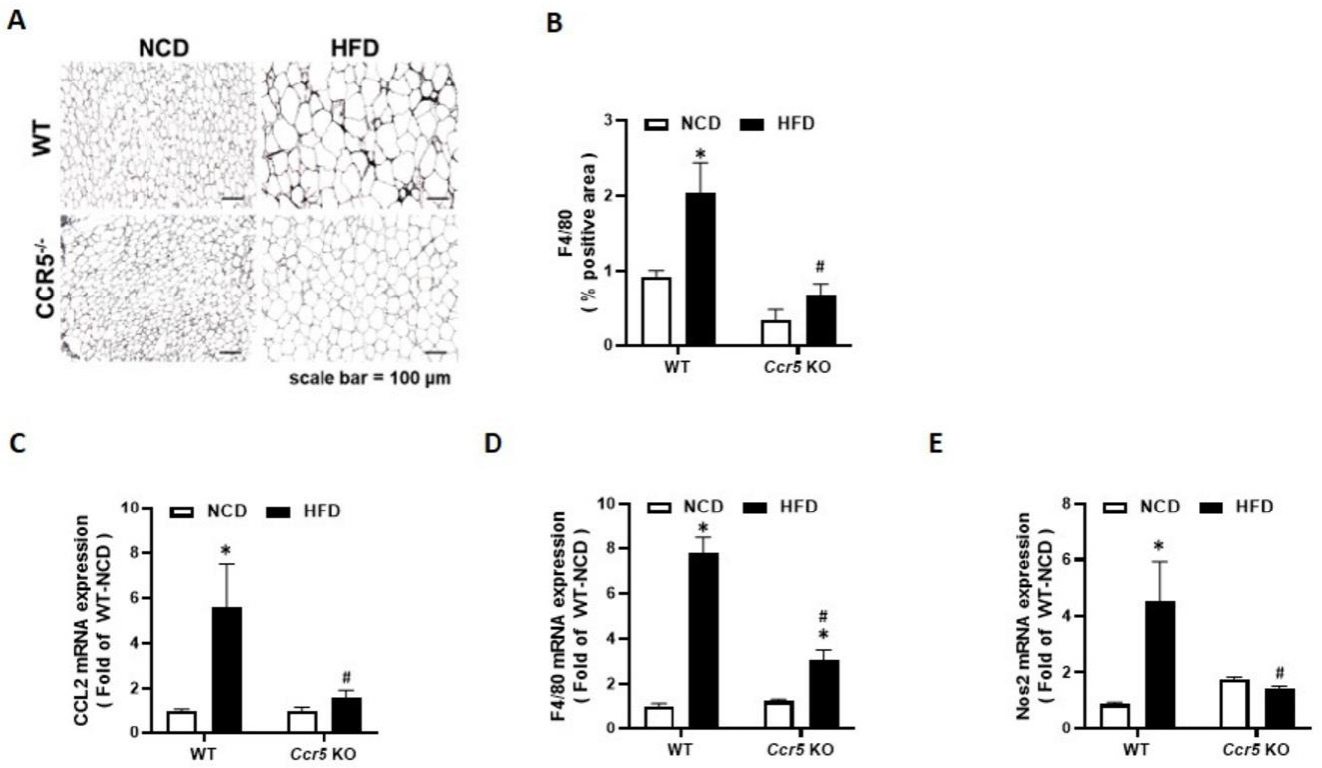
** CCR5 deficiency mice attenuates HFD-induced macrophage infiltration and adipose tissue inflammation.** Mice from each genotype (WT and CCR5-deficient) were fed either a normal chow diet (NCD) or a high-fat diet (HFD) starting at 8 weeks of age for a duration of 12 weeks (n = 6). **(A)** Representative images of epididymal adipose tissue stained for F4/80 to visualize macrophage infiltration. Scale bar = 100 µm. **(B)** Quantification of F4/80-positive area (% positive area). **(C-E)** Relative mRNA expression levels of Ccl2 (MCP-1), F4/80, and Nos2 in epididymal fat measured by qRT-PCR. Data are presented as mean ± SEM. *p < 0.05 (NCD vs. HFD) ; #p < 0.05 (CCR5-deficient mice vs wild-type mice).

Macrophage infiltration into white adipose tissue is a hallmark of obesity-associated inflammation. To elucidate the role of CCR5 in obesity-related adipose inflammation, epididymal fat from wild-type (WT) and CCR5⁻/⁻ mice was analyzed after 12 weeks of normal chow diet (NCD) or high-fat diet (HFD) feeding. Immunohistochemical staining revealed a significant increase in F4/80⁺ macrophage infiltration in the epididymal fat of HFD-fed WT mice compared to NCD-fed controls (Fig. 7A). In contrast, this effect was markedly attenuated in CCR5⁻/⁻ mice. Quantification confirmed that the F4/80-positive area was significantly elevated in WT-HFD mice but substantially reduced in CCR5⁻/⁻-HFD mice (Fig. 7B), suggesting that CCR5 deficiency may mitigate obesity-induced macrophage accumulation. To further characterize adipose tissue inflammation, the expression of key inflammatory and macrophage markers was evaluated by qRT-PCR. The chemokine Ccl2 (MCP-1), which mediates monocyte/macrophage recruitment, was strongly upregulated in HFD-fed WT mice, whereas this induction was significantly diminished in CCR5⁻/⁻ mice (Fig. 7C). Similarly, F4/80 mRNA levels were elevated in WT-HFD mice but significantly lower in CCR5⁻/⁻-HFD mice (Fig. 7D). Furthermore, Nos2 (iNOS), a marker of pro-inflammatory M1 macrophages, was significantly increased in WT-HFD mice but not in CCR5⁻/⁻ mice (Fig. 7E). Collectively, these findings indicate that CCR5 deficiency may protect against obesity-induced adipose tissue inflammation.

## Discussion

3T3-L1 preadipocytes are widely utilized for studying adipocyte differentiation. Melloni's research has shown that these cells exhibit high levels of RANTES secretion, a ligand for CCR5 10. To minimize the influence of endogenous RANTES, we opted to use 3T3-F442A preadipocytes as our model to investigate the regulatory role of CCR5 activation in adipogenesis. Compared to 3T3-L1 preadipocytes, 3T3-F442A cells exhibit relatively low RANTES expression (data not shown). In this study, we found that CCR5 expression during adipocyte differentiation is time-dependent, with levels increasing in the early stages. These findings suggest that CCR5 may play a significant role in adipocyte differentiation.

Moreover, we demonstrated that CCL5/CCR5 activation stimulates adipocyte differentiation, an effect that can be blocked by the CCR5-specific antagonist Maraviroc. Wu et al. reported an upregulation of both mRNA and protein levels of RANTES and CCR5 in the adipose tissue of obese mice 1. Additionally, obese humans with metabolic syndrome exhibited higher mRNA levels of RANTES and CCR5 in their adipose tissues compared to lean individuals 1. Our* in vitro* study shows that CCR5 activation by RANTES directly regulates adipocyte differentiation. These results align with those of Mello Coelho et al., who observed an increase in CCR5^+^ fat-storing cells within the aging thymus 11. They clarified the relationship between CCR5 and adipocytes, demonstrating that treatment with CCR5 ligands, including CCL3, CCL4, and RANTES, resulted in increased expression of adipocyte differentiation markers PPARγ2 and aP2 in 3T3-L1 cells 11. However, their study did not establish the specificity of CCR5 in adipocyte differentiation. In contrast, our study successfully suppressed RANTES-stimulated adipocyte differentiation through Maraviroc pretreatment. Recently, Yu et al. explored the regulation of ectopic fat deposits in skeletal muscle and found that Vitamin K3 stimulated RANTES expression and release, leading to increased preadipocyte migration and intramuscular fat deposition 12. These effects were inhibited by the CCR5 antagonist Maraviroc, suggesting that the CCL5/CCR5 axis plays a crucial role in regulating intramuscular fat content. Collectively, our findings indicate that CCR5 activation can stimulate adipocyte differentiation and that the RANTES/CCR5 axis may be pivotal in the development of obesity.

Additionally, we demonstrated that RANTES/CCR5 activation promotes adipocyte differentiation through the ERK1/2 signaling pathway. Several studies have suggested that ERK plays a regulatory role in adipocyte differentiation. For instance, Xin et al. showed that depletion of BK channels promotes adipocyte differentiation by activating the ERK pathway 13. Prusty's research indicated that the MEK/ERK signaling pathway affects adipogenesis differently depending on the timing of its activation during the differentiation process 14. Wu's study showed that activating the MEK/ERK pathway enhances adipogenic differentiation in mesenchymal stem cells 15, while repression of MEK/ERK activation suppressed adipogenesis in 3T3-L1 cells 16. Conversely, Ueno's research indicated that activating the MEK/ERK-CREB cascade via prostaglandin F2α suppressed adipogenesis 17. Kim found that (-)-epigallocatechin gallate inhibited adipocyte differentiation through the MEK/ERK pathway 18. These findings highlight the importance of ERK signaling in adipocyte differentiation, and the discrepancies may relate to the different roles ERK plays at various stages of adipogenesis 19.

In our *in vivo* study, we observed elevated plasma RANTES levels and adipose CCR5 expression in WT mice fed a high-fat diet (HFD) compared to those on a normal chow (NC) diet. These findings are consistent with Wu's observations in humans 1, 20, suggesting that the RANTES/CCR5 system is highly activated in obesity. Additionally, we found that CCR5 deficiency significantly ameliorated HFD-induced obesity in mice. While our *in vivo* observations align with our *in vitro* results, they differ from Kitade's findings, which reported no significant differences in body weight and adipose tissue weight between CCR5^-/-^ and WT mice on either NC or HFD 4. This discrepancy may be due to different methods used to establish CCR5 knockout mice. Other studies support our findings; for example, Huh et al. reported that HFD-fed mice treated with a dual CCR2/5 antagonist had significantly reduced body weight gain and less expansion of adipocyte size compared to HFD-fed controls 21. Another study indicated that RANTES recruits subcutaneous preadipocytes, promoting intramuscular fat deposition in obese mice 22. Clinical observations also align with our findings, with Huber et al. and Baturcam et al. demonstrated significant increases in circulating RANTES levels as well as RANTES and CCR5 expression in the adipose tissue of obese individuals compared to lean ones 20, 23. Moreover, physical exercise significantly reduced the expression of both RANTES and CCR5 in the adipose tissue of obese subjects, alleviating obesity-related complications 20. Collectively, our findings and these observations support the notion that the RANTES/CCR5 axis plays a crucial pathogenic role in obesity development.

## Conclusion

**Figure 8:**
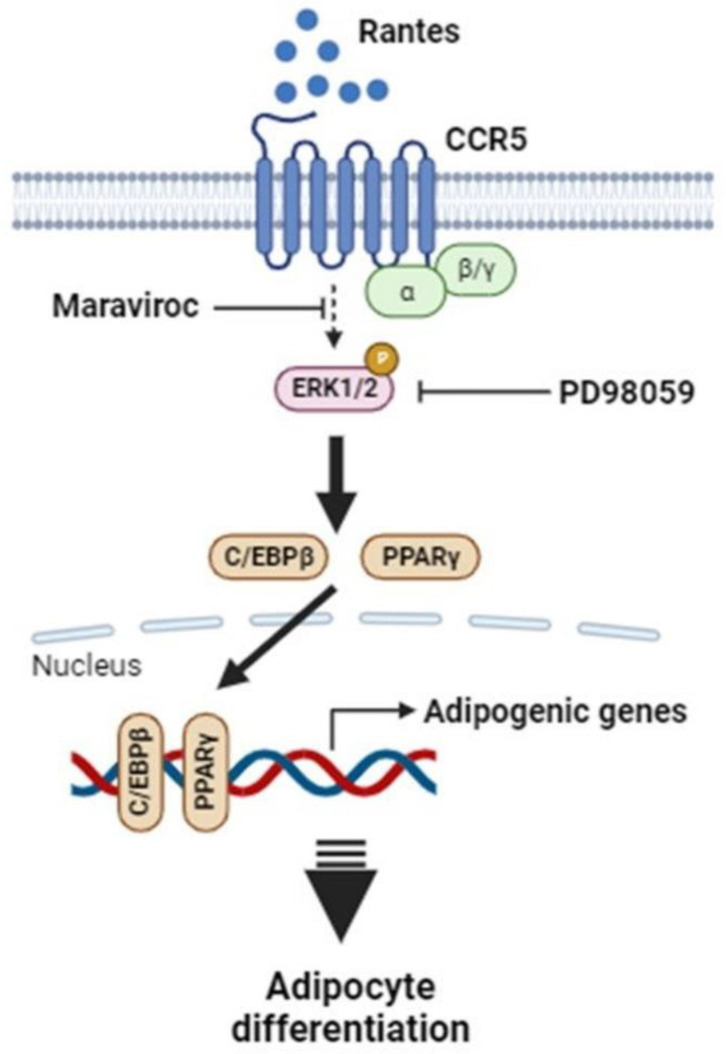
** Proposed schematic outlining the mechanism by which elevated RANTES-induced CCR5 activation stimulates adipocyte differentiation via the ERK1/2-dependent pathway.** Abbreviations: PPARγ, peroxisome proliferator-activated receptor γ; C/EBPs, CCAAT/enhancer-binding proteins; ERK1/2, extracellular regulated kinase 1/2.

In summary, this study demonstrates that RANTES promotes adipocyte differentiation through CCR5 activation and the ERK-dependent pathway. Adipose RANTES and CCR5 levels are elevated in HFD-induced obese mice compared to lean controls, and CCR5 deficiency mitigates HFD-induced obesity. These findings underscore the significant role of RANTES/CCR5 signaling in regulating adipose tissue expansion in obesity. Based on these results, we propose a schematic representation of CCR5 signaling in adipocyte differentiation (Fig. 8). Our results suggest that CCR5 deficiency may offer potential benefits in preventing obesity and its related metabolic disorders by improving adipose hypertrophy. Targeting RANTES/CCR5 signaling in adipose tissue may be a promising strategy for preventing obesity and alleviating its associated complications.

## Figures and Tables

**Table 1:** Effects of Diet and CCR5 Knockout on Body Weight, Adipose Tissue Weight, and Metabolic Parameters in Mice

	Wild-type mice		CCR5 knock out mice	
Diet	NCD	HFD	NCD	HFD
Food intake (g/mouse/day)	3.24±0.24	1.89±0.14*	3.39±0.16	2.03±0.17*
Energy intake (kcal/mouse/day)	9.78±0.96	9.65±0.72	10.24±0.93	10.31±0.88
Body weight (g)	25.33±0.56	46.16±1.98*	26.43±1.27	37.04±1.30*^#^
Body weight gain	3.8±0.58	20.32±5.33*	3.48±0.91	12.94±2.16*^#^
Epi weight (g)	0.54±0.12	2.33±0.27*	0.73±0.15	1.93±0.36*^#^
Sub weight (g)	0.30±0.08	2.05±0.67*	0.37±0.10	1.06±0.13*^#^
BAT weight (g)	0.10±0.02	0.31±0.14*	0.14±0.04	0.21±0.04*^#^
Fasting glucose (mg/dL)	101.60±7.89	168.80±18.38*	99.80±5.63	135.00±17.39*^#^
Fasting insulin (μU/mL)	4.89±2.50	16.81±5.31*	6.29±2.02	9.29±3.80*^#^
HOMA-IR	1.73±0.73	7.04±2.52*	1.43±0.75	3.06±1.56^#^
CCR5 mRNA expression level	1.00±0.26	2.87±1.55*	N.D.	N.D.
CCL5 mRNA expression level	1.00±0.36	2.04±0.22*	1.24±0.33	3.46±1.49*^#^

Note: normocaloric diet (NCD) or high fat diet (HFD), epididymal fat weight (Epi weight), subcutaneous fat weight (Sub weight), brown fat weight (BAT weight), homeostatic model assessment for insulin resistance (HOMA-IR), not detected (N.D.). Food intake refers to the average daily amount of food consumed (g/mouse/day). Energy intake (kcal/mouse/day) was calculated by multiplying the daily food intake by the caloric density of each diet: NCD = 3.02 kcal/g, HFD = 5.1 kcal/g. Energy intake = Food intake × kcal/g. Data are means±S.D. (n = 6). Statistics: Two-way analysis of variance followed by post-hoc analysis with Tukey's multiple comparisons test. *P < 0.05 versus NCD; #P < 0.05 versus WT
